# Comparison of Cardiovascular Outcomes and All-Cause Mortality Between Patients With and Without Epilepsy: A Systematic Review and Meta-Analysis of Observational Studies

**DOI:** 10.7759/cureus.54706

**Published:** 2024-02-22

**Authors:** Azrung Fayaz, Mehnahil Raza, Areeba Khan, Pranav Mohandas, Hailegiorgis Getnet Ayalew, Prinka Perswani, Calvin R Wei, Mahmoud Ahmed Abdelbaki

**Affiliations:** 1 Internal Medicine, College of Physicians and Surgeons, Peshawar, PAK; 2 Internal Medicine, Hayatabad Medical Complex Peshawar, Peshawar, PAK; 3 Medicine, King Edward Medical University, Lahore, PAK; 4 Critical Care Medicine, United Medical and Dental College, Karachi, PAK; 5 Medicine, Tbilisi State Medical University, Tbilisi, GEO; 6 Medicine, Saint Paul's Hospital Millennium Medical College, Addis Ababa, ETH; 7 Internal Medicine, Liaquat University of Medical and Health Sciences, Hyderabad, PAK; 8 Research and Development, Shing Huei Group, Taipei, TWN; 9 Medicine, Military Medical Academy, Cairo, EGY

**Keywords:** systematic review, myocardial infarction, all-cause mortality, cardiovascular outcomes, epilepsy

## Abstract

This meta-analysis aimed to assess the all-cause mortality and cardiovascular outcomes among patients diagnosed with epilepsy. The entire process of this systematic review and meta-analysis adhered to the Preferred Reporting Items for Systematic Reviews and Meta-Analyses (PRISMA) guidelines to ensure transparency and reporting completeness. A comprehensive search strategy was employed to identify relevant studies in electronic databases, including PubMed, Embase, and the Cumulative Index of Nursing and Allied Health Literature (CINAHL), from January 1, 2010, to January 15, 2024. Outcomes assessed in this meta-analysis included all-cause mortality, cardiovascular mortality, stroke, myocardial infarction, and arrhythmias. A total of 12 studies were included in this meta-analysis with a pooled sample size of 7,026,313. The majority of included studies were conducted in Taiwan (n=4). Our study revealed that individuals with epilepsy faced a higher risk of all-cause mortality, cardiovascular mortality, and stroke. Although there was a higher incidence of myocardial infarction and arrhythmias among epilepsy patients, this disparity did not reach statistical significance. There is a need for future research to explore the impact of epilepsy types, antiepileptic drugs, and lifestyle factors on cardiovascular outcomes.

## Introduction and background

Epilepsy impacts over 50 million individuals globally [1], with developed countries showing an overall prevalence ranging from 5% to 8% [2]. It is characterized by recurrent seizures arising from synchronous abnormal or excessive neural activity in the brain [3]. Epilepsy remains a multifaceted challenge, not only due to the disruptive episodic seizures but also because of the accompanying disabilities and social stigma experienced by patients. From a public health perspective, this issue carries significant medical, sociological, cultural, and economic ramifications [2]. Individuals with epilepsy face a two- to threefold increased risk of premature mortality compared to the general population [4]. A substantial proportion of this heightened risk, approximately 15%, is attributable to sudden cardiac death (SCD) or acute myocardial infarctions [5]. This elevated risk can be partially attributed to a higher prevalence of comorbid cardiac conditions and an increased burden of cardiovascular risk factors among people with epilepsy. For example, individuals with epilepsy are more likely to be obese, physically inactive, and smokers, and they exhibit a worse cardiovascular risk profile compared to the general population [6].

Individuals diagnosed with epilepsy face heightened morbidity and mortality rates associated with ischemic heart disease and cerebrovascular ailments [7]. The interplay between cardiovascular disease and epilepsy is intricate. Verrier et al. introduced the concept of the "epileptic heart" to elucidate alterations occurring in both the myocardium and coronary vasculature due to recurrent catecholaminergic surges and hypoxemia accompanying seizure activity in chronic epilepsy patients [8]. These repeated cardiotoxic insults are thought to induce mechanical and electrical dysfunction, consequently elevating the risk of adverse cardiovascular events like arrhythmias and SCD. There is speculation regarding the potential overlap between the latter and sudden unexpected death in epilepsy (SUDEP) [8]. Moreover, epilepsy can serve as a symptom of cardiovascular disease. Adult-onset epilepsy is frequently attributed to stroke [9-10], while seizures later in life may indicate underlying subclinical cerebrovascular issues.

Currently, there is a scarcity of research focused on investigating cardiovascular outcomes such as mortality, arrhythmias, and cardiac arrest in individuals with epilepsy, despite their significant clinical relevance. Therefore, to enhance our understanding of the epidemiology of cardiovascular disease and its outcomes in this population, we conducted a meta-analysis to assess the all-cause mortality and cardiovascular outcomes among patients diagnosed with epilepsy.

## Review

Methodology

Study Search

The entire process of this systematic review and meta-analysis adhered to the Preferred Reporting Items for Systematic Reviews and Meta-Analyses (PRISMA) guidelines to ensure transparency and reporting completeness. A comprehensive search strategy was employed to identify relevant studies in electronic databases, including PubMed, Embase, and the Cumulative Index of Nursing and Allied Health Literature (CINAHL), from January 1, 2010, to January 15, 2024. The search was conducted using a combination of Medical Subject Headings (MeSH) terms and keywords related to epilepsy, cardiovascular disease, and incident vascular events. The search was conducted by two authors independently, and any disagreement between the two authors was resolved through discussion. We limited our search to studies that were published in the English language.

Study Selection

The initial screening of titles and abstracts was conducted independently by two reviewers to identify potentially eligible studies. Subsequently, a full-text review was performed to assess the suitability of each study based on predefined inclusion and exclusion criteria. Discrepancies between reviewers were resolved through discussion or consultation with a third reviewer when necessary. The final selection of studies was based on the following inclusion and exclusion criteria: (a) studies comparing outcomes between epilepsy and patients without epilepsy and (b) studies conducted in adults (age >18 years). We excluded studies that lack a control or comparison group. We also excluded case reports, case series, non-original studies, and reviews.

Data Extraction

Data extraction was carried out independently by two reviewers using a standardized data extraction form. The extracted information included study characteristics (e.g., author name, publication year, study region, sample size), participant demographics (e.g., age, gender, comorbidities), and outcomes. Outcomes assessed in this meta-analysis included all-cause mortality, cardiovascular mortality, stroke, myocardial infarction, and arrhythmias. Any discrepancies in data extraction will be resolved through consensus or consultation with a third reviewer.

Quality Assessment

The methodological quality of the included studies was assessed using established tools such as the Newcastle-Ottawa Scale for observational studies. Two independent reviewers evaluated each study's quality based on predefined criteria, including study design, sample size, and outcome assessment. Disagreements between reviewers were resolved through discussion or consultation with a third reviewer when needed. Studies were categorized as low, moderate, or high quality based on their overall score, and this quality assessment was considered in the interpretation of results.

Data Analysis

Quantitative synthesis of the data was performed using RevMan 5.4.1. A pooled effect size, such as risk ratios (RR), was calculated along with their corresponding 95% confidence intervals (CI) to compare dichotomous outcomes between patients with and without epilepsy. Heterogeneity among studies was assessed using the I-square statistic, and a random-effects model will be employed if significant heterogeneity is observed (I-square: >50%). Meta-regression was done using all-cause mortality as an outcome variable to determine the potential causes of heterogeneity. Publication bias was assessed using Egger's test performed using Stata Statistical Software: Release 17.0 (2021; StataCorp LLC, College Station, Texas, United States).

Results

**Figure 1:**
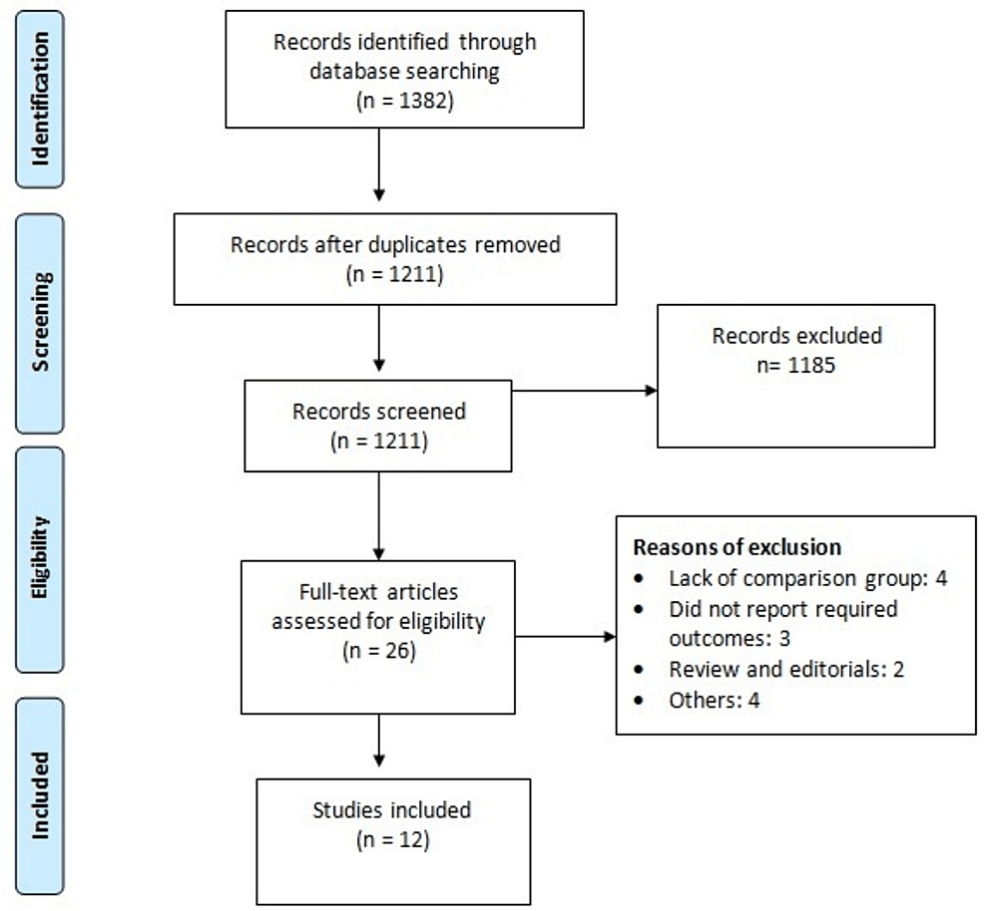
PRISMA flowchart of study selection PRISMA: Preferred Reporting Items for Systematic Reviews and Meta-Analyses

Through online database searching, we identified 1382 studies. After removing duplicates, we initially screened 1211 records. The full text of 26 studies was retrieved, and a detailed evaluation was done based on the predefined inclusion and exclusion criteria. Finally, 12 studies were included in this meta-analysis [11-22]. Figure 1 shows the process of study selection. Table 1 shows the characteristics of the included studies. The pooled sample size was 7,026,313, among which 1,696,076 individuals were diagnosed with epilepsy, accounting for 24% of the total. The majority of included studies were conducted in Taiwan (n=4) [12-14,16] followed by the United States (n=3) and the United Kingdom (n=2) [11,21-22] and Canada (n=2) [15,19]. Table 2 shows the quality assessment of all included studies.

**Table 1:** Characteristics of the included studies

Author	Year	Region	Number of patients with epilepsy (n)	Number of patients without epilepsy
Badheka et al. [11]	2010	United States	6	492
Chang et al. [12]	2014	Taiwan	3812	15,248
Cheng et al. [13]	2021	Taiwan	5411	21,644
Hsu et al. [14]	2019	Taiwan	6746	26,984
Husein et al. [15]	2021	Canada	751	42,128
Lin et al. [16]	2019	Taiwan	891	891
Mayer et al. [17]	2023	United Kingdom	682,349	682,349
Olesen et al. [18]	2011	Denmark	4,560,114	29,205
Shah et al. [19]	2023	Canada	7220	494,676
Wang et al. [20]	2023	United Kingdom	2699	326,733
Wannamaker et al. [21]	2015	United States	21,035	16,636
Wilson et al. [22]	2018	United States	39,203	39,090

**Table 2:** Quality assessment of the included studies using the Newcastle-Ottawa Scale

Author	Selection	Comparability	Assessment	Overall
Badheka et al. [11]	3	2	2	Good
Chang et al. [12]	3	2	4	Good
Cheng et al. [13]	2	2	3	Good
Hsu et al. [14]	3	1	3	Good
Husein et al. [15]	3	2	4	Good
Lin et al. [16]	2	2	3	Good
Mayer et al. [17]	3	1	4	Good
Olesen et al. [18]	2	1	2	Fair
Shah et al. [19]	2	2	4	Good
Wang et al. [20]	3	2	2	Good
Wannamaker et al. [21]	3	2	3	Good
Wilson et al. [22]	3	2	3	Good

All-Cause Mortality and Cardiovascular Mortality

**Figure 2:**
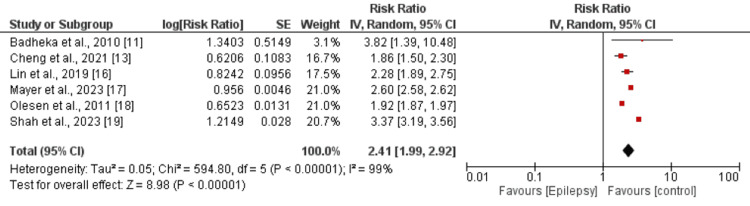
Risk of all-cause mortality References: [11,13,16-19]

**Figure 3:**
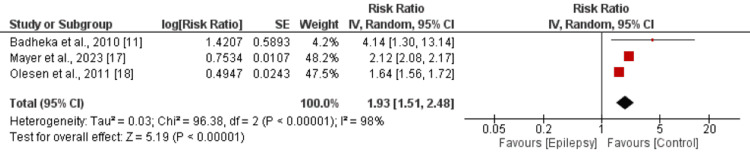
Risk of cardiovascular mortality References: [11,17,18]

Six studies compared the risk of all-cause mortality between patients with and without epilepsy. As depicted in Figure 2, the risk of all-cause mortality was significantly higher in patients with epilepsy compared to those without epilepsy (RR: 2.41, 95% CI: 1.99-2.92). There was significant heterogeneity among the study results (I-square: 99%). Table 3 presents the results of the meta-regression that none of the variables was proven to be significant with all-cause mortality. Three studies evaluated the risk of cardiovascular mortality between the two groups, revealing a significantly greater risk in epilepsy patients compared to controls (RR: 1.93, 95% CI: 1.51-2.48) as shown in Figure 3. Significant heterogeneity was also observed among these study results (I-square: 98%).

**Table 3:** Meta-regression taking all-cause mortality as an outcome variable Statistical test: Wald test *: significant at a p-value of <0.05

Variables	All-cause mortality	
	Coefficient	P-value
Age	0.019	0.095
Number of males	0.0001	0.408
Number of individuals with diabetes	0.0002	0.645
Number of individuals with hypertension	0.0005	0.154
Number of Individuals with dyslipidemia	0.0015	0.115
Year of publication	0.03	0.27
Country of publication	-0.023	0.793

Secondary Outcomes

**Figure 4:**
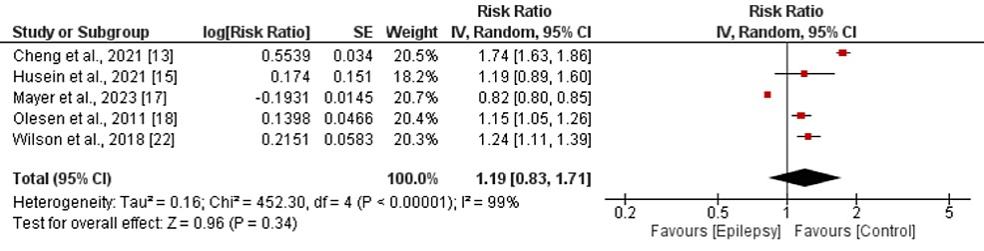
Comparison of myocardial infarction References: [13,15,17,18,22]

**Figure 5:**
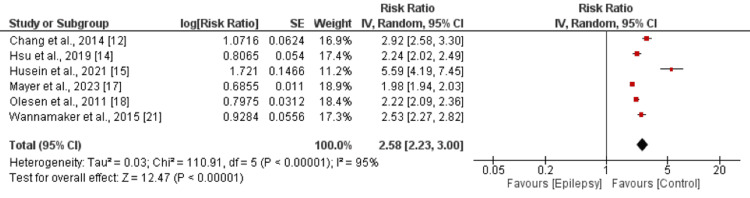
Comparison of stroke References: [12,14,15,17,18,21]

**Figure 6:**
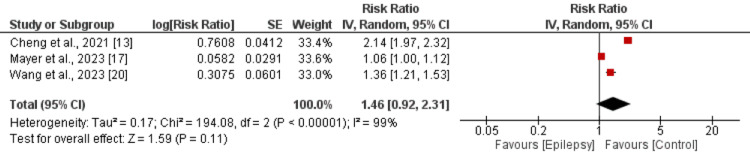
Risk of arrhythmias References: [12,17,20]

The risk of myocardial infarction was higher in patients with epilepsy, but the difference was not significantly different between patients with epilepsy and without epilepsy (RR: 1.19, 95% CI: 0.83-1.71) as shown in Figure 4. Significant heterogeneity was reported among the study results (I-square: 99%). On the other hand, the risk of stroke was significantly higher in patients with epilepsy compared to their counterparts (RR: 2.58, 95% CI: 2.23-3.00) as shown in Figure 5. Significant heterogeneity was reported among the study results (I-square: 95%). The risk of developing arrhythmias was higher in patients with epilepsy, but the difference was not significantly different between patients with epilepsy and those without epilepsy (RR: 1.46, 95% CI: 0.92-2.31) as shown in Figure 6. Significant heterogeneity was reported among the study results (I-square: 99%).

Discussion

This meta-analysis aimed to compare the risk of mortality and cardiovascular events between patients with epilepsy and those without. Our study revealed that individuals with epilepsy faced a higher risk of all-cause mortality, cardiovascular mortality, and stroke. Although there was a higher incidence of myocardial infarction and arrhythmias among epilepsy patients, this disparity did not reach statistical significance. Findings presented at the annual meeting of the American Academy of Neurology showed that individuals with status epilepticus had a greater prevalence of cardiovascular risk factors and a heightened susceptibility to cardiovascular diseases compared to those without this condition [23].

The cross-sectional analysis of the epilepsy group demonstrated significant differences in cardiovascular risk factors, outcomes, and poorer lifestyle behaviors [13,15,17]. These factors likely contribute to the elevated incidence rates of cardiovascular outcomes. Despite adjustments for patients with prior stroke and vascular risk factors, the risk of cardiovascular disease remained elevated in other observational cohorts, suggesting that epilepsy-related factors also play a role in adverse cardiovascular outcomes [15,24]. 

Our meta-analysis revealed an elevated risk of stroke among patients with epilepsy. It is likely that a common pathophysiological mechanism, such as atherothrombosis, contributes to this association. Additionally, there appears to be a link between cardiovascular risk factors, such as cholesterol levels and left ventricular hypertrophy, and the risk of developing epilepsy later in life [25]. Moreover, patients with epilepsy demonstrate a higher prevalence of cardiovascular risk factors and elevated levels of markers indicating oxidative stress compared to the general population [26].

In our analysis, we observed a significantly higher prevalence of comorbidities, including hypertension, diabetes, and dyslipidemia, in individuals with epilepsy. Previous research suggests that the presence of these comorbidities contributes to an increased risk of cardiovascular events and all-cause mortality in people with epilepsy [27]. Similarly, a recent systematic review of population-based cohort studies found that patients with epilepsy have a greater burden of vascular risk factors and are at higher risk of stroke and myocardial infarction compared to those without epilepsy [28].

Interestingly, patients with seizures but no prior history of vascular disease were found to have an increased risk of myocardial infarction, albeit to a lesser extent than their risk for subsequent stroke [18,22]. This indicates that unexplained epilepsy may represent an overall risk factor for vascular diseases, not limited solely to stroke. Furthermore, vascular comorbidities are more commonly found in individuals with epilepsy than in control subjects, suggesting an excess of vascular risk factors in this population [21]. Additionally, a cross-sectional study revealed that patients with late-onset epilepsy (≥55 years) and no history of stroke exhibit a higher prevalence of hypercholesterolemia and left ventricular hypertrophy compared to controls [29].

The pooled analysis indicated an increased likelihood of arrhythmias among individuals with epilepsy. This correlation between epilepsy and arrhythmias is further substantiated by epidemiological inquiries. For instance, a cross-sectional investigation drawing data from 67,786 patients documented by a general practitioner registry in the Netherlands examined comorbidities across various cardiovascular ailments. Results revealed that 26.5% of patients had received diagnoses of at least one cardiovascular condition, with 10.5% exhibiting two or more. Notably, the most notable association identified within cardiovascular diseases was between heart failure and arrhythmias. Moreover, the most robust association spanning both cardiovascular and noncardiovascular conditions was observed between arrhythmias and epilepsy [29].

Grasping the clinical significance of the association between epilepsy and subsequent vascular diseases, as elucidated in this review, presents challenges. It's essential to deliberate whether unexplained seizures could be considered a vascular risk factor, meriting deeper examination and intervention. They could potentially function as "transient ischemic attack (TIA) equivalents," aiding in the identification of individuals at elevated risk of subsequent vascular events. Notably, existing evidence indicates that these individuals encounter a heightened stroke risk akin to the probability of a first clinically evident stroke in those with silent brain infarction [30]. As a result, screening these patients for vascular risk factors emerges as a prudent course of action

However, this meta-analysis has several limitations. Firstly, we were unable to explore whether specific seizure types or epilepsy syndromes are associated with an elevated risk of particular cardiovascular events due to insufficient data in the included studies. Understanding differences among seizure types may offer insights into varying cardiovascular risks among epilepsy patients. Secondly, we couldn't assess the influence of antiepileptic drugs (AEDs) on cardiovascular outcomes. Some AEDs may have cardiovascular side effects, and elucidating these associations can inform medication choices for epilepsy patients, especially those with higher cardiovascular risk. Additionally, it's important to acknowledge that the majority of studies included in this meta-analysis were conducted in Taiwan, which may limit the generalizability of our findings to other populations. Lastly, there is considerable heterogeneity in all assessed outcomes in this meta-analysis, possibly stemming from variations in sample size, study location, design, and participant baseline characteristics. Future research should investigate the role of lifestyle factors in the relationship between epilepsy and cardiovascular outcomes. Factors such as physical activity, diet, and stress management may affect both conditions, and interventions targeting these factors warrant exploration.

Clinical Implications

In clinical practice, understanding the link between epilepsy and vascular diseases is paramount for the accurate diagnosis and optimal management of patients. Patients with epilepsy who also present with vascular risk factors or comorbidities may require tailored treatment approaches to address both conditions effectively. For instance, clinicians should prioritize cardiovascular risk assessment and implement preventive measures to mitigate the risk of vascular events in individuals with epilepsy. Moreover, awareness of this association can aid in the early identification of vascular-related complications in epilepsy patients, enabling timely intervention and improved clinical outcomes.

## Conclusions

Our meta-analysis, comprising 12 studies, underscores a heightened risk of all-cause mortality, cardiovascular mortality, and stroke in individuals with epilepsy compared to those without. Although the risk of myocardial infarction and arrhythmias appeared elevated, statistical significance was not reached. Understanding the clinical implications of epilepsy as a vascular risk factor warrants further investigation. However, limitations in data availability and heterogeneity across studies highlight the need for future research to explore the impact of epilepsy types, AEDs, and lifestyle factors on cardiovascular outcomes.
